# Fluorescent Clade IIb Lineage B.1 Mpox Viruses for Antiviral Screening

**DOI:** 10.3390/v17020253

**Published:** 2025-02-13

**Authors:** Francisco Javier Alvarez-de Miranda, Rocío Martín, Antonio Alcamí, Bruno Hernáez

**Affiliations:** Centro de Biología Molecular Severo Ochoa, Consejo Superior de Investigaciones Científicas (CSIC), Universidad Autónoma de Madrid (UAM), 28049 Madrid, Spain

**Keywords:** poxvirus, fluorescent reporter, mpox, antiviral, clade IIb, bisbenzimide

## Abstract

The ongoing global outbreak of mpox caused by clade IIb viruses has led to more than 100,000 confirmed cases around the world, highlighting the urgent need for antiviral research to combat current and future mpox outbreaks. Reporter viruses expressing fluorescent proteins to monitor viral replication and virus spreading in cell culture provide a powerful tool for antiviral drug screening. In this work, we engineered two recombinant mpox clade IIb viruses by inserting, under the control of the vaccinia early/late promoter 7.5, the coding sequence of two different fluorescent proteins (EGFP and TurboFP635) in a previously unreported location within the viral genome. These recombinant viruses replicate in BSC-1 cells at rates similar to those of the parental virus. We show how these reporter mpox viruses allow the discrimination of infected cells by cell flow cytometry and facilitate the quantification of viral spread in cell culture. Finally, we validated these reporter viruses with two previously known inhibitors of poxvirus replication, cytosine arabinoside (AraC) and bisbenzimide.

## 1. Introduction

Since the eradication of smallpox in 1980, the emergence of a new human poxvirus to fill the niche left by the variola virus has become a constant threat. The mpox virus (MPXV) is a member of the orthopoxvirus genus and is the causative agent of the zoonotic mpox disease, characterized by fever, lymphadenopathy and a vesiculopustular rash that resembles smallpox [1,2].

The MPXV was first reported in human populations in the 1970s, with two clearly differentiated lineages: virulent clade I (endemic to central Africa), with a mortality rate of 10%, and clade II (endemic to west Africa), causing a milder disease with a mortality rate of <3.6% [3,4,5]. Mpox was historically transmitted to humans as a zoonosis from rodents and primates [6], causing little human transmission and sporadically escaping to non-endemic regions in traveler-related outbreaks caused by strains belonging to clade II [7,8]. However, the recent resurgence of this disease in 2022 was marked by increased human-to-human transmission, causing around 91,000 confirmed cases in 115 countries between January 2022 and October 2023 [9,10] and leading to the World Health Organization (WHO) declaring this disease a public health emergency of international concern (PHEIC) for almost one year. In summer 2024, human transmission of a new clade Ib was detected, leading the WHO to declare a second PHEIC due to the expanded distribution of this clade in central and eastern Africa [11]. The 2022 global outbreak and the current alarm raised by clade Ib highlight the necessity of new research tools to improve diagnosis, surveillance and public health interventions against virulent poxviruses affecting humans [12,13].

Genomic analysis indicates that the global outbreak strains that emerged in 2022 grouped into a new clade IIb lineage B. This new lineage derives from an outbreak (clade IIb lineage A.1) occurring in Nigeria between 2017 and 2018 [14,15] and encompasses 51 unique single-nucleotide changes relative to this previous strain. The large clinical and epidemiological differences between endemic and global MPXVs have brought interest to the new global strain, with recent studies focusing on its specific biological differences and its potential adaptations to the human host [16,17].

In order to visualize viral infections and facilitate research, several recombinant orthopoxviruses encoding reporter molecules have been generated in the past [18,19,20]. In the specific case of MPXV, previous studies have generated reporter MPXVs based on older lineages to study this virus. Insertion of the GFP-coding sequence between the genes OPG103 and OPG104 or the firefly luciferase-coding sequence between OPG56 and OPG57 was used to study MPXV clade I intravenous infection in cynomolgus macaques [21], CAST/EiJ mice and African dormice [22]. In addition, two different clade IIa MPXVs encoding firefly luciferase between OPG202 and OPG204 [23,24] or near OPG57 [25] were generated and characterized in BALB/c mice and prairie dogs. In all cases, the encoded reporter proteins helped to characterize the inoculation routes and track viral spread, which helped to advance the characterization of these strains. However, despite the great impact of the 2022 outbreak and the differences between this virus and previous strains, no reporter MPXVs have been generated on a clade IIb lineage B background.

In the context of the global outbreak, these kinds of reporter viruses might be useful to facilitate the development of new antivirals and more effective vaccines against mpox, which still has no specifically approved treatments in the USA [26]. Tecovirimat, a drug developed to treat smallpox and the only antiviral authorized by the European Medicines Agency (EMA) for treating mpox in Europe [27], has been proved to inhibit viral spread within the host and reduce mortality in animals in several preclinical studies [28]. However, despite its relative efficacy, early treatment with tecovirimat is sometimes limited by availability and regulatory approvals in different regions, and other antivirals used for severe cases, like brincidofovir and cidofovir, come with severe side effects [29]. In addition, several cases of resistance to tecovirimat were quickly detected during the 2022 PHEIC [30]. This resistance was mostly associated with single amino acid changes in the MPXV F13 protein, the target of tecovirimat, and other studies have reported that single point mutations are sufficient to confer resistance [31,32]. MPXV lineage B shows a much larger mutation rate than that of lineage A, higher than the ~0.35 nucleotide substitutions per year previously predicted for this virus [33]. So, we cannot rule out the possibility of new resistant MPXV strains arising due to sustained human-to-human transmission and exposure to the available treatments.

In this work, we generate and characterize two new reporter MPXVs based on the emergent clade IIb lineage B. We show how these MPXVs, expressing fluorescent proteins, constitute valuable biological tools for primary research on this emerging virus and analyze their suitability for the rapid testing of new antiviral candidates.

## 2. Materials and Methods

### 2.1. Cells and Viruses

BSC-1 cells (ATCC CCL-26, African green monkey renal epithelial cells) were grown at 37 °C, 95% humidity and 5% CO_2_ in Dulbecco’s modified Eagle medium (DMEM, Gibco, Grand Island, NY, USA) supplemented with 5% FBS, 2 mM L-glutamine and antibiotics.

The MPXV clade IIb lineage B.1 was isolated during the 2022 outbreak in Madrid and later plaque-purified and sequenced (European Nucleotide Archive accession number GCA_964276875). MPXV stocks were produced following our previously described protocol for other orthopoxviruses [34]. All the procedures involving the MPXV were performed at the biosafety level 3 laboratory (BSL3) at Centro de Biologia Molecular Severo Ochoa (CBM).

### 2.2. Generation of Recombinant MPXVs

The generation of the MPXV2b-EGFP and MPXV2b-FP635 viruses was achieved through homologous recombination to insert the fluorescent-protein-coding sequences into the intergenic region between the MPXV2b loci OPG185 and OPG187. For MPXV2b-EGFP, we generated the plasmid pSH-EGFP by cloning flanking sequences consisting of approximately 380bp of the intergenic region at each side of the EGFP-coding sequence under vaccinia virus promoter P7.5 (GenBank accession number: X55811.1 [35]) by In-Fusion cloning (Takara, Shiga, Japan), using the following oligonucleotides: flanking—left fragment: 5′-ggtatcgataagcttttgacttacataaatatctgggat-3′ and 3′-aagggtactggatcctatatacccaagtaatgcattaggt-5′; flanking—left vector: 5′-ggatccagtacccttcacct-3′ and 3′-aagcttatcgataccgtcgacctc-5′; flanking—right fragment: 5′-aattctgcagatatcagacctacctctcttaaaatggttg-3′ and 3′-gcggtggcggccgcttatgatctgtcaatgaaataccatt-5′; flanking—right vector: 5′-agcggccgccaccgcggtgg-3′ and 3′-gatatctgcagaattgtcttgaccc-5′. In the case of MPXV2b-FP635, we generated the plasmid pSH-FP635 by substituting the EGFP-coding sequence in pSH-EGFP with the far-red fluorescent protein TurboFP635-coding sequence [36] with the oligonucleotide FP635_fragments: 5′-gtagatcataacaccatggtgggtgaggatagcg-3′ and 3′-attgtcttgacccttttagctgtgccccagtttgc-5′; FP635_vector: 5′aagggtcaagacaattctgc-3′ and 3′-ggtgttatgatctacttccttaccg-5′.

Next, we developed an infection/transfection protocol to promote the recombination between the plasmid and the virus. BSC-1 cells in 6-well plates were sequentially infected with MPXV clade IIb lineage B1 for 1 h and then transfected with the corresponding recombinant plasmid using Fugene HD (Invitrogen, Carlsbad, CA, USA). When the cytopathic effect was complete, the cells were harvested and used as an inoculum to infect at a low multiplicity of infection (MOI) in semi-solid carboxy-methyl cellulose (CMC) DMEM. Green or red plaques were isolated under the fluorescence microscope. Finally, the recombinant viruses MPXV2b-EGFP and MPXV2b-FP635 were purified by 4 consecutive rounds of plaque assay.

### 2.3. Viral DNA Extraction and MPXV Whole-Genome Sequencing

To obtain viral DNA from the recombinant viruses generated, infected cells were treated with a nuclease mix (DNAsa I, Micrococcal Nuclease S7 and RNAsa A; Roche, Basel, Switzerland) to eliminate unenveloped genetic material, and viral DNA was released from the viral particles after proteinase K (Invitrogen, Carlsbad, CA, USA) treatment. The viral DNA was then isolated after phenol–chloroform extraction. The presence of viral DNA was confirmed by PCR with specific oligonucleotides for MPXV OPG204: 5′-tatgccggccatatgatagacatcgaaaatgaaatcaccg-3′ and 3′-agggataggcttaccctccaatactactgtagttgtaag-5′. Contamination with bacterial DNA was ruled out by PCR with oligonucleotides targeting the 16S ribosomal RNA gene: 27F: 5′-agagtttgatcmtggctcag-3′, 1492R: 3′-tacggytaccttgttacgact-5′.

After viral DNA isolation, libraries for Illumina sequencing were prepared using the Illumina DNA Prep Tagmentation kit following the manufacturer’s instructions, and NGS was performed in a MiniSeq system (Illumina, San Diego, CA, USA) using a 2 × 150 run with the MiniSeq Mid Output kit (300 cycle) (Illumina, San Diego, CA, USA). The obtained reads were mapped against the corresponding expected reference poxvirus genome with Bowtie v2.5.4 aligner software using default parameters and visualized using the Integrative Genomics Viewer (IGV). The resulting coverage for both viral genomes was above 4000×, and the corresponding sequence analysis ruled out the presence of additional and non-desirable mutations in the MPXV2b-EGFP and MPXV2b-FP635 genomes. Files containing the raw reads were deposited in the ENA (European Nucleotide Archive) under the project number PRJEB82673.

### 2.4. Plaque Assay

To determine viral titers, ten-fold serial dilutions of viral stocks or samples were used to inoculate BSC-1 cell monolayers. After 90 min adsorption at 37 °C, the viral inoculum was replaced with semi-solid CMC DMEM supplemented with 2% FBS. At 72 h post-infection (hpi), the cells were fixed in 4% formaldehyde for 20 min at room temperature, and plaques were stained with 0.1% (*w*/*v*) crystal violet.

### 2.5. Flow Cytometry

The day before the assay, 3 × 10^4^ BSC-1 cells per well were seeded in 24-well plates (ThermoFisher, Waltham, MA, USA). The next day, the cells were infected with the indicated doses of MPXV2b-EGFP or MPXV2b-FP635. One hour after infection, the inoculum was removed and replaced with fresh DMEM 2% FBS. At 24 hpi, the cells were scraped and transferred to a V-bottom 96-well plate, washed twice with PBS and fixed with 4% paraformaldehyde (PFA) for 15min at RT before exiting the BSL-3 facilities. Then, infected cells were discriminated using an AURORA 4L (Cytek, Fremont, CA, USA) spectral cytometer. In order to account for autofluorescence during infection, BSC-1 cells were infected with a non-fluorescent MPXV clade IIb lineage B.1 with the same MOI and used as negative control for the spectral unmixing. The results were analyzed with FlowJo v10.9.0 software.

### 2.6. Viral-Driven Fluorescence in Microplate Reader

The day before the assay, 8 × 10^3^ BSC-1 cells per well were seeded in 96-well clear/black-bottom plates (ThermoFisher, Waltham, MA, USA). The next day, the cells were infected with the indicated doses of MPXV2b-EGFP or MPXV2b-FP635. One hour post-infection, the inoculums were removed and replaced with DMEM 2% FBS or the indicated doses of 1-beta-D-arabinofuranosylcytosine (AraC, Sigma-Aldrich, Merck, Germany) or bisbenzimide Hoechst 33342 (H42, Sigma-Aldrich, Merck, Germany) diluted in medium. At the indicated times post-infection, the plates were washed with PBS and fixed with 4% PFA for 15 min at room temperature before exiting the BSL-3 facilities. Fluorescence was measured in a FLUOstar Omega (BMG Labtech, Ortenberg, Germany) plate reader at 485/520 nm or 584/620 nm for MPXV2b-EGFP and MPXV2b-FP635, respectively. Each experiment was performed in at least three technical replicates. To check cell toxicity due to AraC and H42, the cell viability was determined after incubation with those concentrations tested using a Cell Titer 96 Aqueous One Solution cell proliferation assay (Promega, Madison, WI, USA), following the manufacturer’s indications.

## 3. Results

### 3.1. Generation of Fluorescent Clade IIb MPXVs

**Figure 1:**
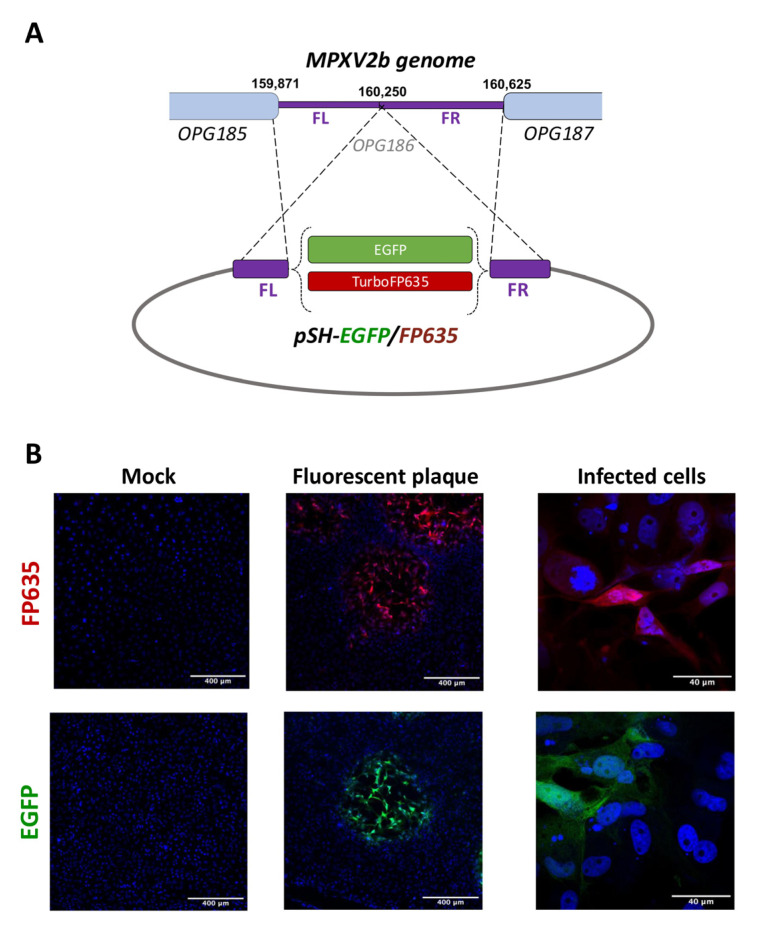
Fluorescent clade IIb lineage B.1 MPXVs. (**A**) Schematic representation of the recombination process for the generation of MPXV2b-EGFP and MPXV2b-FP635. (**B**) BSC-1 cells were infected at 0.1 pfu/cell with MPXV2b-EGFP or MPXV2b-FP635 and screened under the fluorescence microscope at 48 hpi. FL: flanking left, FR: flanking right.

For the generation of fluorescent MPXVs, we inserted the coding sequence for EGFP or TurboFP635 into the genome of an MPXV clade IIb lineage B.1 isolated during the 2022 outbreak in Madrid. The insertion site chosen was the intergenic region between MPXV genes encoding glycoproteins 162 and 163 (OPG185 and OPG187 in the poxvirus genome, respectively (Figure 1A)). This novel insertion site contains no open reading frames, is around 750 bp long and corresponds to the vaccinia virus (VACV) Copenhagen A57R pseudogene (OPG186). After screening green or red plaques under the fluorescence microscope (Figure 1B), the recombinant viruses MPXV2b-EGFP and MPXV2b-FP635 were purified and fully sequenced to ensure that they had not undergone other unexpected mutations. In addition, all the plaques remained fluorescent after four sequential passages on BSC-1 cells, ensuring the stability of the genomic modifications performed.

**Figure 2:**
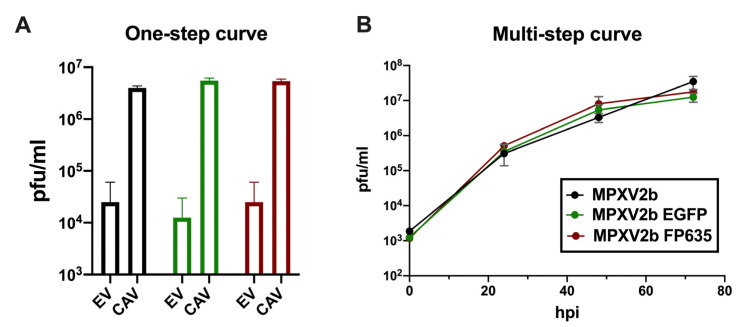
In vitro replication of MPXV2b-EGFP and MPXV2b-FP635. (**A**) BSC-1 cells were infected with the indicated viruses at 10 pfu/cell for the one-step growth curve. Virus production was determined by plaque assay at 24 hpi from fractions containing cell-associated viruses (CAV or extracellular viruses (EV). (**B**) BSC-1 cells were infected with the indicated viruses at 0.05 pfu/cell for the multi-step growth curve. Total virus production was determined by plaque assay at the indicated times.

To assess whether the replication in cell culture was affected by the insertion of the fluorescent proteins, the BSC-1 cells were infected with MPXV2b-EGFP or MPXV2b-FP635 at a high MOI (10 pfu/cell) to determine the production of cell-associated virus and extracellular virus at 24 hpi. In both cases, the comparison with the parental virus showed no significant differences (Figure 2A). Similar results were obtained after the infection of cells at a low MOI (0.05 pfu/cell) and on the determination of total virus yields at several times post-infection (Figure 2B). These results demonstrated that the insertion of EGFP- or TurboFP635-coding sequences in the intergenic region between OPG185 and OPG187 did not affect the production of viral progeny in cell culture.

### 3.2. Rapid Detection and Discrimination of Infected Cells by Tracking Fluorescence

**Figure 3:**
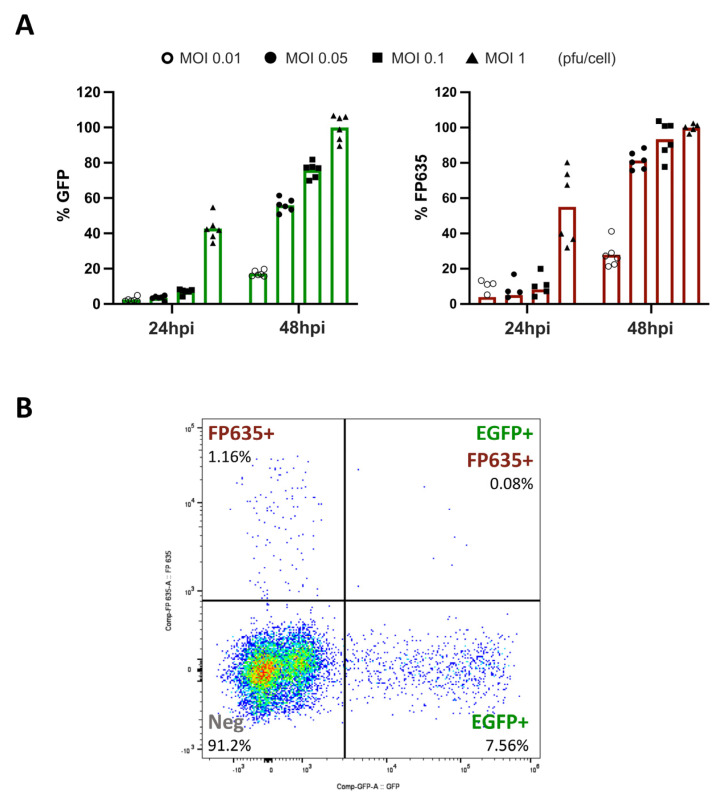
Detection of infected cells by viral-driven fluorescence. (**A**) BSC-1 cells were infected at different MOIs with MPXV2b-EGFP or MPXV2b-FP635. Viral-driven fluorescence, relative to the mean fluorescence intensity at 48 hpi MOI 1 pfu/cell, was measured at the indicated times. Minimum of 5 replicates for each condition. (**B**) BSC-1 cells were co-infected with MPXV2b-EGFP and MPXV2b-FP635 at MOI 0.1 and 0.01 pfu/cell, respectively. At 24 hpi, cells were fixed, and infected cell populations were discriminated by flow cytometry.

Next, we analyzed how the expression of fluorescent proteins allowed for the rapid quantification of infected cells. For this, BSC-1 cells in 96-well plates were infected with increasing MOIs (ranging from 0.01 to 1 pfu/cell), and viral-driven fluorescence was measured at 24 and 48 hpi (Figure 3A). This allowed the rapid analysis of the viral spread over time at different virus concentrations, observing maximum fluorescence at 48 hpi when infecting with MOI 1 and 0.1 pfu/cell.

Then, we assayed the possibility of discriminating infected cells during co-infection with both fluorescent viruses. BSC-1 cells were co-infected with MPXV2b-EGFP and MPXV2b-FP635 using MOIs 0.1 pfu/cell and 0.01 pfu/cell, respectively, to differentiate the number of fluorescent cells after infection with different viral doses. At 24 hpi, the number of cells expressing EGFP or FP635 was determined by cell flow cytometry (Figure 3B). The infected cell populations observed were consistent with the initial infectious doses tested. In addition, a small population of co-infected cells simultaneously expressing EGFP and FP635 was detected, indicating co-infection progression.

### 3.3. Fluorescent Viruses Ease the Detection of Antiviral Activities Against MPXV2b

**Figure 4:**
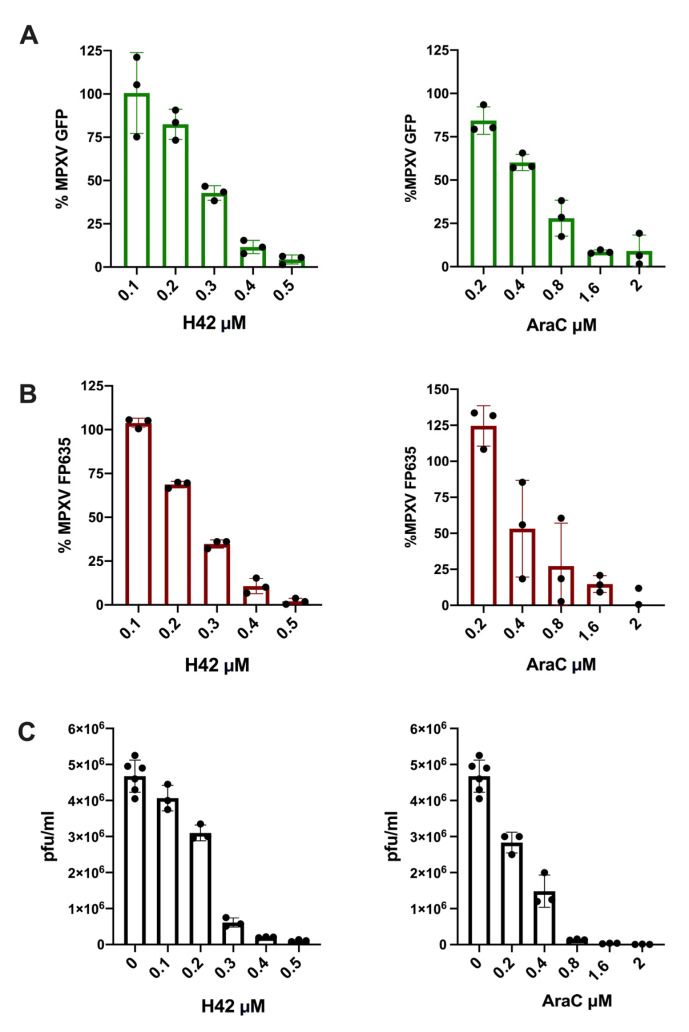
Fluorescent MPXVs can ease the detection of antiviral activity. (**A**) BSC-1 cells were infected with MPXV2b-EGFP (MOI 0.1 pfu/cell) and treated with serial dilutions of H42 or AraC. Viral-driven fluorescence at 48 hpi, relative to the mean fluorescence of untreated control, is shown. (**B**) BSC-1 cells were infected with MPXV2b-FP635 (MOI 0.1 pfu/cell) and treated with serial dilutions of H42 or AraC. Viral-driven fluorescence at 48 hpi, relative to the mean fluorescence of untreated control, is shown. (**C**) BSC-1 cells were infected with MPXV2b (MOI 0.1 pfu/cell) and treated with the previously assayed concentrations of H42 or AraC. Viral titers at 48 hpi, measured by plaque assay, are shown. All the experiments were performed using at least triplicates.

Finally, we tested the suitability of fluorescent clade IIb lineage B1 MPXVs to quickly evaluate candidate molecules with antiviral properties. Two previously known inhibitors of poxvirus replication were used: AraC and H42 [37]. Following our previous results for the detection of infected cells, BSC-1 cells were infected at MOI 0.1 pfu/cell and then treated with serial dilutions of the antiviral compounds. After allowing the infection to progress for 48 h, the fluorescence was evaluated in a microplate reader. A dose-dependent decrease in viral-driven fluorescence after the treatments with H42 or AraC was observed for both MPXV2b-EGFP (Figure 4A) and MPXV2b-FP635 (Figure 4B). Then, to confirm that the observed decrease in fluorescence was representative of antiviral activity, the effect of the drugs in the viral progeny was evaluated by plaque assay. We observed a steady decrease in viral titers consistent with the observed changes in viral-driven fluorescence for the same conditions (Figure 4C). These results show that fluorescent viruses can be effectively used to quickly evaluate compounds with antiviral activity against MPXV clade IIb lineage B1.

## 4. Discussion

After more than forty years since the eradication of smallpox, the lack of vaccination and exposure to poxviruses has caused a decrease in protective immunity and an unprecedented susceptibility to poxviral infections in the global population. In this context, the 2022 mpox PHEIC evidenced the need to develop better surveillance, treatments and preventive drugs against poxviruses.

In this work, we generated two fluorescent clade IIb lineage B.1 MPXVs as new tools for antiviral research against the global MPXV strain. We selected the intergenic region between OPG185 and OPG187 as the insertion site for our fluorescent proteins. This region was chosen due to the lack of coding regions and the sufficient distance from nearby genes, as it corresponds to the pseudogene OPG186. Since the insertion of fluorescent proteins in this locus did not affect replication in vitro, it could be useful for the generation of new reporter MPXVs. Clade I MPXVs also lack OPG186, including the new lineage clade Ib that has recently gained global attention and led to the WHO declaring a second mpox PHEIC [12,38]. This emerging clade, characterized by its higher transmissibility and potential for severe clinical outcomes, is causing human-to-human transmission in the Democratic Republic of the Congo and surrounding countries but also causes sporadic cases outside the endemic regions [11,39].

Additionally, we investigated the potential use of these reporter MPXVs using two previously known poxvirus inhibitors, H42 and AraC, to demonstrate their potential use to screen libraries of molecular compounds with putative antiviral properties against MPXV. Recent studies have found H42 to be a good antiviral against MPXV, a tecovirimat-resistant VACV and other DNA cytoplasmic replicating viruses such as African swine fever virus [40,41], paving the way for the development of new drugs against poxviruses. However, antiviral screening by classical plaque assays is a heavily hands-on process, which is accentuated by MPXV being a strict BSL-3 pathogen. Fluorescent MPXVs appear as valuable research tools that allow the rapid quantification of viral-driven fluorescence, which serves as a reliable estimator of viral spread. Our results prove that the effects of H42 and AraC on fluorescence are consistent with their effects on viral titers and similar to the previously reported half-maximal inhibitory concentrations (IC50s) of these compounds against poxviruses [37,41]. This can ease and accelerate the discovery of new antiviral activities when screening molecule collections.

In addition, despite our experiments being held up by the lack of machinery within BSL-3 containment, this would not be the case in facilities specialized for antiviral testing. The usage of microplate readers or cytometers inside BSL-3 would allow for the continued tracking of viral spread without the need for fixating samples, allowing for even easier and faster experiments. In addition, these reporter MPXVs can also be used to visualize viral infections withing the host, allowing to quickly link disease progression and viral load while offering ethical advantages by reducing the number of animals required.

Finally, the availability of two fluorescent viruses that are easy to differentiate can help the development of new methods for mutant virus generation. The generation of recombinant viruses is an invaluable tool for basic research in poxviruses, helping to characterize their functions [42]. A new method for the rapid generation of VACV recombinants using CRISPR/Cas9 technology was developed by using two different fluorescent VACVs [43,44]. Adapting this method for MPXV could allow the rapid generation of MPXV recombinants, facilitating research on this virus.

The fluorescent MPXVs described here will facilitate studies to characterize the new clade IIb lineage B and the development of antivirals against this emergent MPXV clade.

## Data Availability

Sequencing data are available at the European Nucleotide Archive repository (ENA, https://www.ebi.ac.uk/ena/browser/home, accessed on 20 January 2025) in project number PRJEB82673.
